# Evaluation of G × E × M Interactions to Increase Harvest Index and Yield of Early Sown Wheat

**DOI:** 10.3389/fpls.2020.00994

**Published:** 2020-07-10

**Authors:** Kenton Porker, Michael Straight, James Robert Hunt

**Affiliations:** ^1^Crop Sciences, Agronomy Group, South Australia Research and Development Institute, Urrbrae, SA, Australia; ^2^School of Agriculture, Food & Wine, Waite Research Institute, The University of Adelaide, Urrbrae, SA, Australia; ^3^FAR Australia, Mulwala, NSW, Australia; ^4^CSIRO Agriculture and Food, Canberra, ACT, Australia

**Keywords:** winter wheat, defoliation, management, plant density, vernalization

## Abstract

Harvest index (HI) is the ratio of grain to total shoot dry matter and is as a measure of reproductive efficiency. HI is determined by interactions between genotypes (G), environment (E), and crop management (M). Historic genetic yield gains due to breeding in wheat have largely been achieved by increasing HI. Environmental factors are important for HI and include seasonal pattern of water supply and extreme temperatures during crop reproductive development. Wheat production in Australia has been dominated by fast-developing spring cultivars that when sown in late-autumn will flower at an optimal time in early spring. Water limited potential yield can be increased by sowing slower developing wheats with a vernalization requirement (winter wheat) earlier than currently practiced such that their development is matched to environment and they flower at the optimal time. This means a longer vegetative phase which increases rooting depth, proportion of water-use transpired, and transpiration efficiency by allowing more growth during winter when vapour pressure deficit is low. All these factors can increase biomass accumulation, grain number and thus grain yield potential. However higher yields are not always realized due to a lower HI of early sown slow developing wheats compared to fast developing wheats sown later. Here, we evaluate genotype × management practices to improve HI and yield in early sown slow developing wheat crops using 6 field experiments conducted across south eastern Australia from 2014 to 2018 in yield environments ranging from ~1 to ~4.7 t/ha. Practices included low plant densities (30–50 plants/m²), mechanical defoliation, and deferred application of nitrogen fertilizer. Lower plant densities had similar yield and HI to higher plant densities. Defoliation tended to increase HI but reduce yield except when there was severe stem frost damage. Deferring nitrogen had a variable effect depending on starting soil N and in crop rainfall. All management strategies evaluated gave variable HI and yield responses with small effect sizes, and we conclude that none of them can reliably increase HI in early sown wheat. We propose that genetic improvement is the most promising avenue for increasing HI and yield in early sown wheat, and postulate that this could be achieved more rapidly through early generation screening for HI in slow developing genotypes than by crop management.

## Introduction

Wheat production in Australia is dominated by fast-developing spring cultivars. For over a century wheat breeding programs have been selecting for faster developing cultivars to escape drought and high temperatures (Davidson et al., 1985; Eagles et al., 2009), and growers have been sowing progressively earlier since the widespread adoption of no-till farming in the 1990s (Anderson et al., 2016). Consequently, flowering times of commercial crops have become earlier for genetic and management reasons, and are currently optimal in many environments (Flohr et al., 2017). In southern Australia, the optimal flowering period is defined by a relatively narrow period in which the combined damage from lack of radiation, frost, drought, and heat are minimized. Optimal flowering periods differ for each ecological zone depending on climate and generally occur during the first half of spring (late August to mid-October). Moving crop flowering progressively closer to this period has led to sustained increases in harvest index (HI) and water-use efficiency and has helped maintain farm yields despite declining water-limited potential yields (Hochman et al., 2017). Fast developing spring wheats are typically sown in late autumn (early May) to flower during this optimal period. However, recent research has demonstrated that water limited potential yield can be further increased by sowing winter or slow-developing spring wheats earlier than currently practiced such that they still flower at the optimal time but have a longer vegetative phase (Hunt et al., 2019). This can only reliably be achieved with cultivars with an obligate vernalization requirement, i.e., winter habit (Penrose and Martin, 1997; Fischer, 2011; Hunt, 2017; Hunt et al., 2019) sown in mid-autumn (April). Winter genotypes have previously been overlooked by growers, agronomist, and breeders due to later sowing in evaluation and agronomy trials and the Genetic (G) × Environment (E) × Management (M) opportunities to maximize yield have not been fully explored. A new generation of winter cultivars have been released in Australia from 2016 onward suitable for planting before 20 April (Hunt et al., 2019). While our understanding of genetic controls of vernalization (Trevaskis, 2010) and importance of achieving optimal flowering times (Flohr et al., 2017; Flohr et al., 2018b) has improved, crop management, and yield physiology of early sown winter cultivars has received little attention.

Due to their vernalization requirement and early sowing, winter cultivars spend longer in the vegetative phase compared to spring cultivars sown later. This means more leaves and potential tillering sites are initiated, and a lengthening of the growing period has the potential to increase water use due to greater rooting depth and thus soil water extraction. It may also increase the proportion of water-use transpired; and transpiration efficiency by allowing more growth during winter when vapour pressure deficit is low (Flohr et al., 2020). All these factors increase dry matter (DM) accumulation, grain number, and thus potential grain yield (GY). However, experiments conducted by Gomez-Macpherson and Richards (1995) and in reviewed experiments of others (Batten and Khan, 1987; Connor et al., 1992) found that GYs of slow developing cultivars were only equivalent to faster developing cultivars sown later despite similar or greater DM in early sown cultivars due to a lower HI. In some instances, yields of early sown slow developing wheats are less than spring wheats due to a lower HI and lodging (Stapper and Fischer, 1990; Riffkin et al., 2003). This presents an opportunity to improve yields in early sown slow developing cultivars by improving HI.

There is scope to improve the HI of early sown wheat. HI is the ratio of the yield of grain to the total shoot DM and can be used as a measure of reproductive efficiency (Donald and Hamblin, 1976). Environmental factors are important determinants of HI and include seasonal pattern of water supply and extreme temperatures during crop reproductive development. One plausible explanation for a reduced HI in early sown slow developing cultivars is related to pattern of water use. In glasshouse experiments, the ratio of pre- and post-anthesis water use has been demonstrated to be strongly related to HI (Passioura, 1977). When established early, winter wheats have a long duration of pre-anthesis growth, and in water-limited environments can use too much water before anthesis such that HI is low compared with spring wheats established later (Gomez-Macpherson and Richards, 1995). Deferred water use trades off against the higher DM accumulation of early established winter cultivars which drives high grain number and produces water-soluble carbohydrates (WSCs) that can be translocated to grain, such that yield of early established winter wheats is at least equivalent to faster spring cultivars established later.

The second explanation is that increased plant height and more leaves lead to competition for carbohydrates between the developing spike and elongating stem of early sown crops (Gomez-Macpherson and Richards, 1995). Genetic improvement is one approach to remove these limitations. Since the green revolution, improvement in wheat GY in many environments has been due to an increase in the number of grains per unit area and HI (Siddique et al., 1989; Hay, 1995; Sayre et al., 1997; Shearman et al., 2005; Flohr et al., 2018a). Increasing the sink size or improved partitioning to spike growth may provide a pathway for the improvement of the HI of wheat (Foulkes et al., 2010). While there may be potential to further adjust phasic development to alter the timing and duration of spike development, a recent study by Flohr et al. (2018a) showed that HI improvement was not due to phase duration and suggests other factors are involved such as partitioning traits. Previous studies in many crops have outlined any genetic or management solution that could either increase the grain growth rate or enhance the remobilization of assimilates from vegetative tissues to grains after anthesis usually leads to a higher HI within a crop (Sadras and Connor, 1991; Yang et al., 2000; Kemanian et al., 2007; Fletcher and Jamieson, 2009; Zhang et al., 2012).

Crop management also has the capacity to modify the pattern of water use and biomass partitioning. There are many examples where variation in HI are mainly attributed to differences in crop management (Kemanian et al., 2007; Peltonen-Sainio et al., 2008). While delayed sowing is a management factor known to increase HI, this is counterproductive for slow developing cultivars as they will flower outside of the optimal window, produce less DM, and have reduced yield potential. Management factors that can improve HI and yield from an early sowing date (prior to April 20) have not yet been explored.

Over supply of nitrogen (N) early in crop development stimulates vigorous vegetative growth and can lead to water deficit in later reproductive phases. Excessive vegetative growth induced by excessive N is commonly known to lower GY and HI in water limited environments and is associated with reduced post-anthesis carbon assimilation in response to a lack of soil water (Van Herwaarden et al., 1998). The water deficit also contributes to poor re-translocation of pre-anthesis reserves. Deferring nitrogen inputs until after the start of stem elongation in winter types therefore has the potential to conserve water use and manage early biomass production. To the best of our knowledge there are no published experiments that have reported on the effect of N fertilizer timing on crop yield in winter cultivars sown early under Australian dryland conditions.

Reducing plant density has also been proposed as a way of reducing early DM accumulation and improving HI in early established slow developing cultivars (Kirkegaard et al., 2014). Early sown winter cultivars provide additional farming system benefits; a longer vegetative period means more DM is accumulated for forage, and there is more time available for stock to graze before the onset of the reproductive phase (Bell et al., 2015). It is often thought that defoliation could be used as a management technique to reduce vegetative growth and early water use. While this would imply an increase in HI, it also trades off against reduced DM accumulation by anthesis as shown by Kirkegaard et al. (2015). There have been some measured instances of deferral of water use assisting recovery of wheat yield (Virgona et al., 2006; Harrison et al., 2010), but the review of Harrison et al. (2011a) found that defoliation on average reduces GY most likely due to reduced DM accumulation.

To our knowledge, there are no reported factorial experiments combining all management factors such as plant density, nitrogen timing, crop defoliation, and genotype to assess their influence on GY and HI. Here, we evaluate the effect of these management practices on HI and GY of four different winter wheat genotypes sown early in two contrasting environments.

## Materials and Methods

### Field Sites

Three field sites were chosen for evaluation of management practices for early sown winter wheat representative of the major medium-low rainfall environments in which wheat is grown in SE Australia (**Table 1**). Experiments were conducted during 2014 and 2015 at Temora and 2017 and 2018 at both Yarrawonga and Loxton. Yarrawonga and Temora have a similar annual rainfall at 470 and 520 mm, respectively, whereas Loxton is considerably drier at 266 mm. Sites will be referred to as Temora 2014 (Te14), Temora 2015 (Te15), Yarrawonga 2017 (Ya17), Yarrawonga 2018 (Ya18), Loxton 2017 (Lo17), and Loxton 2018 (Lo18) from here on. Air temperature was measured at each site using a TGP-4017 TinyTag (Gemini data loggers UK Ltd) temperature logger installed in a radiation screen at a height of 1.2 m. Rainfall was measured with a tipping bucket rain gauge (Tekbox)connected to a Wildeye data logger and telemetry unit.

**Table 1:** Mean annual, summer fallow (Nov–Mar) and growing season (Apr–Oct) rainfall (1984–2018) from nearest Bureau of Meteorology weather station in comparison to rainfall recorded at experimental sites for the relevant growing seasons.

Site	Year	Annual rainfall(mm)	Nov–Mar rainfall (mm)	Apr–Oct rainfall (mm)
Temora	Mean	520	208	312
	2014	436	158	250
	2015	481	169	276
Yarrawonga	Mean	470	197	273
	2017	473	140	276
	2018	336	161	135
Loxton	Mean	266	96	170
	2017	274	120	135
	2018	178	83	92

### Cultivar, Sowing Date, and Crop Management

Winter cultivars were selected based on suitability for early sowing. Prior to the release of new generation winter wheats in 2016, only one cultivar Wedgetail (mid-developing winter) was chosen for Temora in 2014 and 2015. At all other sites three winter cultivars were chosen based on three contrasting development patterns Longsword (fast winter), Kittyhawk (mid-winter), and DS Bennett (mid-slow winter) and planted in mid-April which is optimal for winter cultivars in all environments (**Table 2**). All cultivars have an obligate requirement for vernalization (winter wheat) and weak photoperiod sensitivity.

**Table 2:** Experimental site characteristics including locations, year, coordinates, sowing date, soil mineral N at sowing, genotypes included in experiment, and total N applied.

Site Location/Year (ID)	Coordinates	Sowing Date*	Soil mineral N at sowing (kg/ha), and depth (m)	Genotypes	Total N applied as urea(kg/ha)
Temora 2014 (Te14)	34°43’42”S 147°33’4”E	8 April	86 (1.6)	Wedgetail	100
Temora 2015 (Te15)	34°24’4”S 147°31’12”E	20 April	208 (1.6)		46
Loxton 2017 (Lo17)	34°31’49”S 140°31’59”E	18 April*	48 (0.9)	Longsword Kittyhawk DS Bennett	46
Loxton 2018 (Lo18)	34°30’25”S 140°34’06”E	18 April*	86 (0.9)		46
Yarrawonga 2017 (Ya17)	36°04’04”S 145°54’43”E	13 April*	137 (0.9)		82
Yarrawonga 2018 (Ya18)	36°04’04”S 145°54’43”E	16 April*	47 (0.9),		82

*Sites received supplementary irrigation at sowing.

At all sites if the seedbed was too dry to allow emergence, plots were irrigated with ~10 mm of water applied using pressure compensating drip-line placed in seeding furrows to germinate seed and allow emergence. Seeding depth was approximately 30 mm depending on seed bed moisture. Sowing date is defined as the calendar date at which seeds become imbibed and began the process of germination, i.e., either the date on which they are planted into a moist seed bed, or the date on which they received rainfall/irrigation after being sown into a dry seed bed. In all experiments, chemical fertilizers and pesticides were applied such that nutrient limitations, weeds, pests or diseases did not limit yield. Nitrogen applications were managed according to treatments and the rate depended on site (**Table 2**) average potential yields. Grain protein in all experiments exceeded 11.1% indicating N deficiency was unlikely (Goos et al., 1982; Holford et al., 1992).

All crops were direct-drilled in small plots at Loxton (1.37 m × 7 m) on 228 mm row spacing with press wheels to give six crop rows per plot. Yarrawonga (1.8 m × 15 m) on 225 mm with press wheels to give eight rows per plot. Temora (1.83 m × 10 m) on 305 mm row spacing with press wheels to give six rows per plot.

### Management of Treatments to Manipulate Harvest Index

Management practices were imposed to alter early DM accumulation and partitioning in an attempt to improve HI. Management practices evaluated at all sites included; 1) two nitrogen timings (seedbed N and deferred N) ensuring either adequate N supply at sowing or deferred until early stem elongation (development stage 30–31; Tottman, 1987); two defoliation treatments to simulate grazing (control and defoliation) applied by mechanical mower twice during tillering before DC30; and two plant density treatments (low and high) targeting 50 and 150 plants/m^2^, respectively. Management factors were applied to each cultivar in a factorial fully randomized complete block experiment which equates to eight management combinations per cultivar per site with four replications.

### Measurements

The onset of stem elongation (DC30) was determined by dissection of the main stem on 5 plants at regular intervals using criteria of Tottman (1987). Day of flowering (DC65) was recorded as the date when 50% of the spikes in each plot had at least one visible anther extruded. Total above-ground biomass at maturity (DM) and yield components were estimated by cutting all above ground biomass from a quadrat 0.9 m × 0.5 m (four middle rows from plots) per replicate at maturity (DC89). Plants were cut at ground level and the number of spikes counted to determine the spike density per unit area (SD). A subsample of 50 randomly selected spikes from the quadrat sample were dried at 70°C for 48 hours and threshed by hand and weighed to determine the number of grains per spike (GPS) and grain number per unit area (GN). HI was calculated as the ratio of grain weight to total biomass. Individual grain weight (KW) was measured by weighing 200 grains dried at 70°C for at least 48 h. GYs were measured by machine harvest of the inside four rows of six row plots and are reported at 12.5% moisture content. Harvest grain moisture and grain protein was determined *via* near infrared (NIR) spectroscopy. Plant height (Hght) was measured from the base of stem up until the tip of emerged spike (excluding awns) at physiological maturity. Groundcover was estimated using regular readings of NDVI recorded using a GreenSeeker^®^ (Trimble Inc., Sunnyvale CA).

The severity of reproductive frost stress was estimated by randomly selecting 10 spikes per plot and frost-induced sterility (FIS) was assessed on the outside florets of each spikelet excluding the terminal, basal, and supernumerary spikelets by the method proposed by Martino and Abbate (2019). FIS is the number of sterile florets per spike expressed as a percentage of the total number of possible grains that could have formed in the outside florets. The % of fertile culms (culms with a viable head relative to culms with unviable head) was measured at sites suspected of stem damage from frost.

### Statistical Analysis

Principal component analysis (PCA) was used to interpret and summarize the major patterns of variation due to environment, genotype and management on phenology measurements, yield, and yield components. PCA was calculated based on genotype means for each trait under each environment, to study the inter-relationships among the components conducted using the Unscrambler software (version 10.3, CAMO, Norway). Means were standardized using 1/SD in order to account for the effect of scale.

The effect of all treatments on yield and other parameters were analysed individually using mixed linear models or across environment using ANOVA with site year, cultivar, nitrogen, defoliation, and plant density as factors/fixed effects and block structure as random effects in the statistical package GenStat for Windows (2018) 19th ed. (VSN International Ltd., Hemel Hempstead, UK). Significance is assumed at the 95% confidence level. If management treatment effect sizes were small and explained less than 3% of the variance combined, the factors were pooled (i.e., plant density and nitrogen timing as one management factor) for subsequent analysis and the factorial interactions limited to three-way interactions for interpretations of G × E × M. If the interaction was not significant, then pooled means incorporating the management treatments were used in the comparisons and figures below.

Linear regression between DM and GY was performed on each individual site using GenStat for Windows (2018) 19th ed. (VSN International Ltd., Hemel Hempstead, UK). Data was interpreted in two ways, firstly by comparing the slope and allometric constants (intercept) of genotypes across all E × M combinations. Secondly the deviation (standardized residual) of each variety × management combination from the fitted regression of DM and GY from each site was used in ANOVA to test the significance of the variety and management effect on GY deviations away from the DM/GY regression. For comparison with traditional agronomic analyses, HI of each genotype at each management combination was computed at the plot level.

## Results

### Environment and Flowering Conditions

Summer fallow rainfall at Temora was 40 and 50 mm below average, and growing season rainfall was 62 and 36 mm below average in 2014 and 2015, respectively. The amount and distribution of rainfall at Loxton and Yarrawonga were consistent with long term rainfall averages in 2017. Both sites were considerably drier in 2018 - summer fallow rainfall was close to average being 36 mm less at Yarrawonga, and 13 mm less at Loxton but growing season rainfall was 50% below average at Yarrawonga and 46% at Loxton (**Table 1**). This led to severe spring drought at both sites.

Cold stress events were apparent in all environments, the most severe events occurred at Temora in 2014, and Yarrawonga in 2018 where minimum temperatures reached < −5.0°C in August (**Table 3**). Temperatures below −4°C are likely to cause stem frost damage and occurred at these sites during stem elongation. All sites recorded significant cold events during heading and flowering during September but were least severe at Loxton in 2017. There were few frosts during October at all sites. Heat events were minimal at Yarrawonga in both seasons, however temperatures above 32°C were common at Loxton and in 2017/2018 and Temora in 2014 (**Table 3**). Detailed temperature profiles of Temora 2014 and Yarrawonga 2018 can be found in **Supplementary Figure 1** and **Supplementary Figure 2**.

**Table 3:** Number of days below 0°C (cold stress), lowest recorded minimum temperature and number of days above 32°C (heat stress), and highest recorded maximum temperature during August, September, and October at all environments.

Site	Year	Cold stress events no. of days temperature < −0°C (Min Temperature)			Heat stress events no. of days temperature > 32°C (Max Temperature)		
		Aug	Sep	Oct	Aug	Sep	Oct
Temora	2014	17 (−4.9)	13 (−2.9)	2 (−0.1)	0	0	8 (34.5)
	2015	8 (−3.5)	7 (−2.2)	0	0	0	1 (34.3)
Yarrawonga	2017	11 (−2.5)	5 (−2.4)	1 (−1.3)	0	0	0
	2018	7 (−6.0)	9 (−3.0)	0	0	0	1 (33.3)
Loxton	2017	7 (−1.7)	3 (−1.1)	0	0	1 (32.8)	6 (34.6)
	2018	5 (−3.6)	13 (−3.7)	1 (−1.0)	0	0	7 (36.8

At Loxton and Yarrawonga, flowering time behavior of winter cultivars were consistent across locations and years (**Table 4**), Longsword was 5–10 days earlier than Kittyhawk, and 10–15 days earlier than DS Bennett at Loxton and ~5 days earlier at Yarrawonga. Within cultivars at each environment the flowering time range was small between 2–5 days suggesting there is little effect of management or seasonal conditions on flowering time. At Temora, flowering date was difficult to assess due to stem frost damage. In 2015 undefoliated treatments of Wedgetail flowered on 7 Oct, and defoliated treatments flowered 5–7 days later.

**Table 4:** Range (earliest to latest) in anthesis dates for Longsword, Kittyhawk, DS Bennett, and Wedgetail across all management combinations within environments.

Environment	Longsword	Kittyhawk	DS Bennett	Wedgetail
Loxton 2017	12–16 Sep	20–24 Sep	28 Sep–2 Oct	
Loxton 2018	12–15 Sep	19–24 Sep	2 Oct–7 Oct	
Yarrawonga 2017	5–9 Oct	8–14 Oct	13–14 Oct	
Yarrawonga 2018	3–7 Oct	2–8 Oct	5–10 Oct	
Temora 2015	–	–	–	7 Oct–15 Oct

### Relationship Between DM, GY, and HI

**Figure 1:**
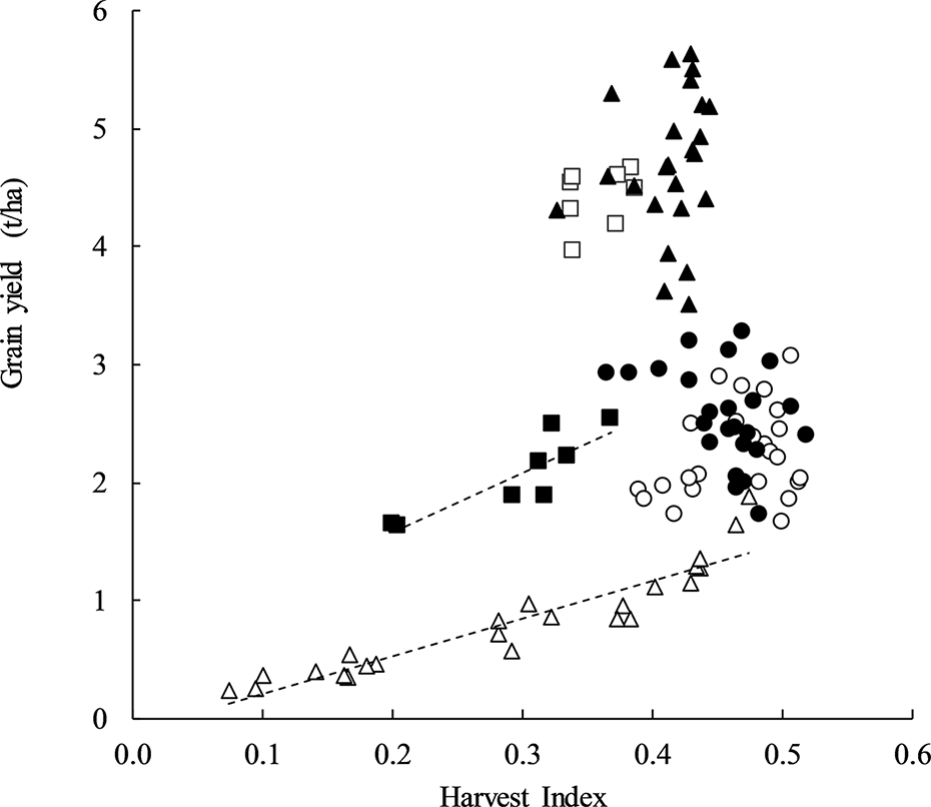
Harvest index plotted against grain yield at Temora 2014 (◾, y = 5.0868x + 0.5564, P = < 0.001, R^2^ = 0.76), Temora 2015 (□, P = ns), Loxton 2017 (●, P = ns), Loxton 2018 (○, P = ns), Yarrawonga 2017 (▲, P = ns), and Yarrawonga 2018 (Δ, y = 3.1897x − 0.1031, P < 0.001, R^2^ = 0.86). Data points are contributed by all of the genetic × management combinations applied to increase HI (n = 112).

There was a strong positive relationship between HI and GY at stem frosted sites Yarrawonga in 2018 and Temora in 2014 explaining up to 86% and 76% of the variation in yield respectively. At other sites, HI was not correlated with GY (**Figure 1**). This means in the absence of severe frost damage a higher HI did not always result in higher GY and specific G × M combinations may be able to achieve a high HI and GY.

**Figure 2:**
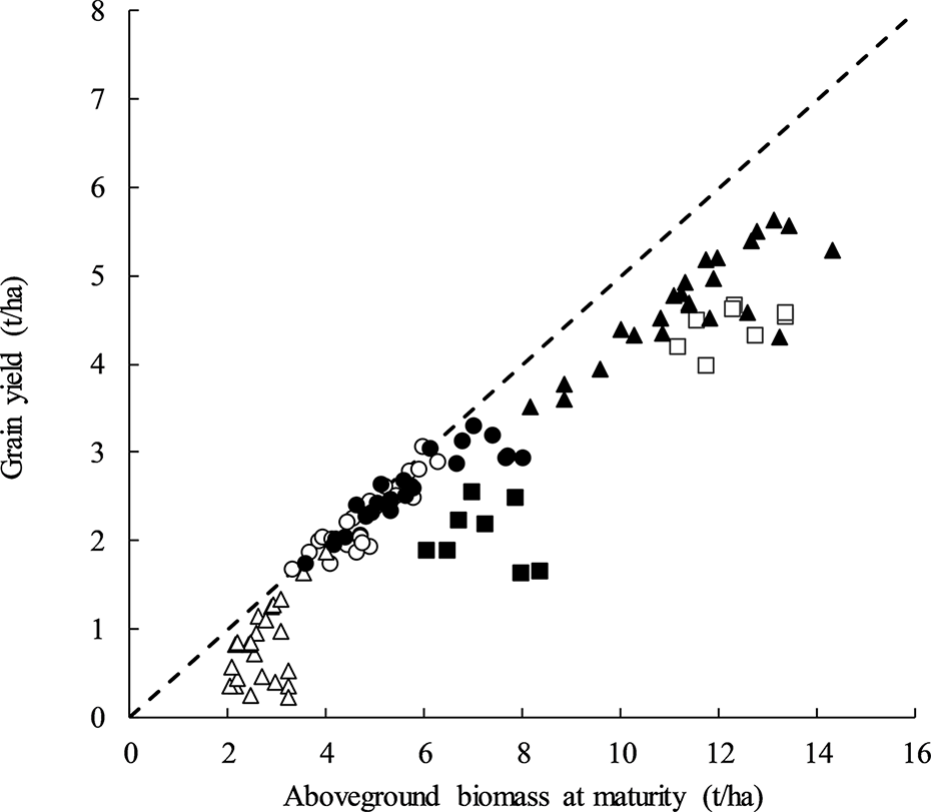
Aboveground biomass at maturity (t/ha) plotted against grain yield (t/ha) at Temora 2014 (■, P = ns) Temora 2015 (□, P = ns), Loxton 2017 (●, y = 0.3021x + 0.8403, P < 0.001, R^2 =^ 0.82), Loxton 2018 (○, y = 0.444x + 0.1014, P <0.001, R^2 =^ 0.79), Yarrawonga 2017 (▲, y = 0.3283x + 0.9569, P < 0.001, R^2 =^ 0.72), and Yarrawonga 2018 (Δ, y = 0.5273x − 0.5928, P < 0.05, R^2^ = 0.34). Data points are contributed by all the management × genetic combinations applied to increase HI (n = 112). The dashed line represents a harvest index of 0.5.

DM and GY were positively associated at all sites except for Temora in both 2014 and 2015 (**Figure 2**). The strong relationship with DM and GY within treatments of similar HI at Yarrawonga in 2017, and Loxton (2017 and 2018) suggest that total biomass can be improved along with maintenance of a high HI using G × M strategies to improve crop yield. The lack of relationship at Temora (2014 and 2015) suggests other factors maybe be driving yield responses.

### Management Interactions on GY, DM, and HI

There was significant variation in GY, DM, and HI across experiments. The largest amount of variation and effect size was due to environment (**Supplementary Table 1**) and while the environment × management factors (seeding density and nitrogen timing) were significant they explained less than 3% of the variance combined and was used to justify pooling plant density and nitrogen timing as one management factor for subsequent analysis.

Site mean GYs ranged from 0.8 t/ha at Yarrawonga in 2018 to 4.7t/ha at Yarrawonga in 2017, DM ranged from 2.7t/ha at Yarrawonga 2018 to 12.3t/ha at Temora in 2015. Site mean HI ranged from 0.29 at Temora 2014 and Yarrawonga 2018 to 0.47 at Loxton in 2018. Higher HI was achieved at Loxton compared to Yarrawonga in both seasons. On average across all experiments defoliation reduced DM by 1.5t/ha and increased HI by 0.05 which resulted in a GY penalty of 0.3 t/ha. Lower density reduced DM by 0.3 t/ha but on average GY and HI were similar between low and high density. Early applied N improved HI by 0.03 and reduced GY by 0.1 t/ha (**Table 5**).

**Table 5:** Mean values for DM at maturity, grain yield (GY), harvest index (HI) in each growing environment, and pooled means for management factors plant density, defoliation, and nitrogen timing.

Factor		Grain yield (t/ha)	DM at maturity (t/ha)	HI
**Environment**	Lo17	2.6^b^	5.7^d^	0.46^a^
	Lo18	2.2^d^	4.8^e^	0.47^a^
	Te14	2.1^c^	7.2^c^	0.29^d^
	Te15	4.4^a^	12.3^a^	0.36^c^
	Ya17	4.7^a^	11.4^b^	0.41^b^
	Ya18	0.8^e^	2.7^f^	0.29^d^
**Plant density**	Low	2.7	7.1^a^	0.39
	High	2.7	7.4^b^	0.38
**Defoliation**	Control	2.8^a^	7.9^a^	0.35^b^
	Defoliated	2.5^b^	6.4^b^	0.40^a^
**Nitrogen timing**	Seedbed N	2.7^a^	6.9^b^	0.40^a^
	Deferred N	2.8^b^	7.4^a^	0.37^b^

Letters a–d indicate means are significantly different at the 95% confidence level.

**Figure 3:**
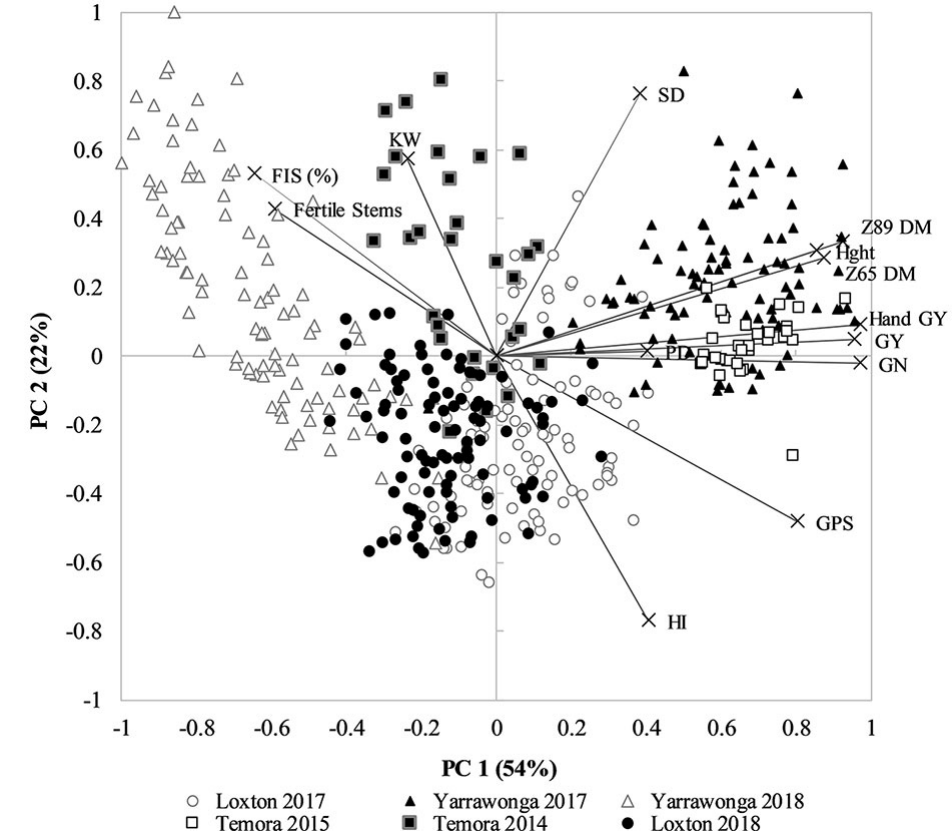
PCA plot of all environment and management combinations for plot grain yield (GY), hand harvest grain yield (hand GY), spike density (SD), plant height (Hght), above ground dry matter at anthesis (Z65 DM), above ground dry matter at maturity (Z89 DM), Harvest Index (HI), Grains per spike (GPS), % sterile florets (%FIS), % Fertile Stems, kernel weight (KW), and grain number (GN). Environments are Temora 2014 (■) Temora 2015 (□), Loxton 2017 (●), Loxton 2018 (○), Yarrawonga 2017 (▲), and Yarrawonga 2018 (Δ). Data points are contributed by the management × genetic combinations applied to increase HI across all replicates (n = 448).

To dissect the relationship between HI, GY, other crop canopy traits and environment an exploratory PCA analysis showed PC1 explained 54% and PC2 22% of the variation in the dataset, which could largely be attributed to environment (**Figure 3**). The strong influence of environment is clear as well as the strong association between GY, GN, Hght, and DM at DC65 and DC89. Importantly, HI was negatively associated to higher sterility (floret and stem), lower kernel weight (KW), and lower spike densities (SD). The PC plot also suggests there is little relationship between HI and GY across environments. HI was only positively associated with GY in environments where frost dramatically reduced the number of grains per spike (GPS) or kernel weight (KW), such as at Yarrawonga in 2018, and Temora 2014 which were associated with a higher % infertile stems, % infertile florets, and lower KW.

The PCA score and loading plot suggest it is possible to achieve both a relatively high HI and GY yield within each environment as directly shown by, **Figure 1** and that this is likely due to management and genetic interventions that are positively correlated to GN and DM.

### G × E × M for Grain Yield, Yield Components and Harvest Index

All genotypes interacted with environment (**Supplementary Table 2**) for GY, DM, HI, GN, SD, and Hght, whereas G × M were limited to a small difference in KW, and the G × defoliation responses depended on environment. Defoliation was the most reliable management strategy to increase HI, however yield responses were still small and variable (including yield reductions) and interacted with environment and cultivar.

**Figure 4:**
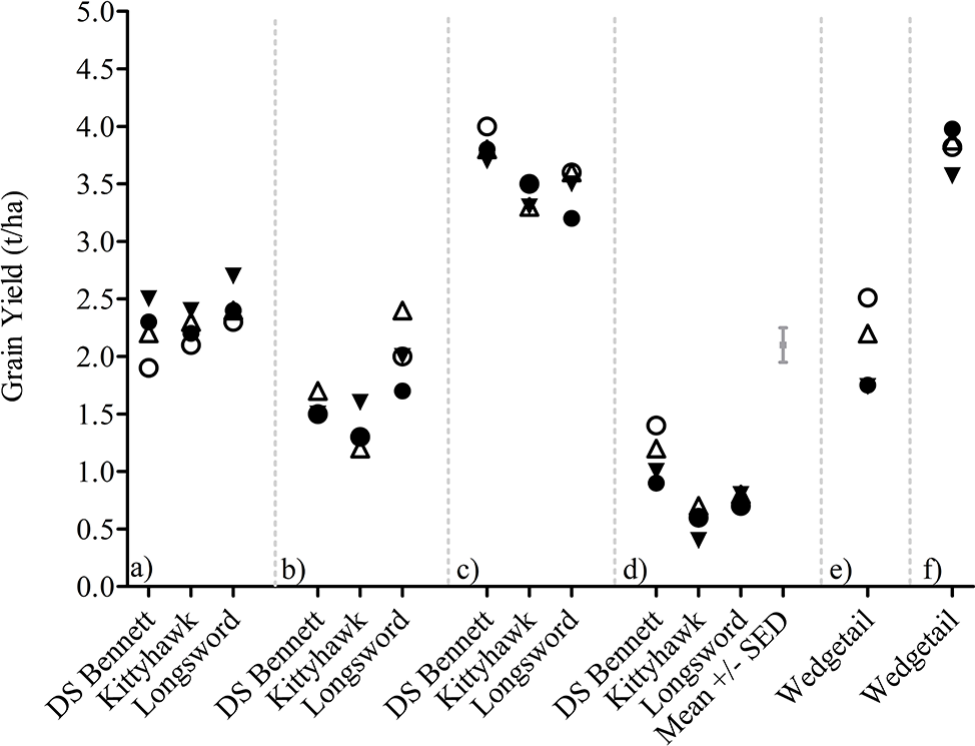
G × E × CM interactions for grain yield at Loxton 2014 **(A)**, Loxton 2015 **(B)**, Yarrawonga 2017 **(C)**, Yarrawonga 2018 **(D)**, Temora 2014 **(E)**, and Temora 2015 **(F)** in genotypes DS Bennett, Kittyhawk, and Longsword. Canopy Management (CM) factors are ▼ Low Density and Seedbed N, Δ Low Density and Deferred N, ● High Density and Seedbed N, and ○ High Density and Deferred N.

Plant density and nitrogen timing effects were small as outlined in *Management Interactions on GY, DM, and HI* and **Supplementary Table 1** and therefore pooled into one management factor titled canopy management (CM). CM interacted with G × E for GY but the G × E × CM interaction was not significant for HI (**Supplementary Table 2**). The yield responses visualized in **Figure 4** demonstrate the variable nature of the yield responses to canopy management strategies and the small effect sizes relative to genotype and environment. There is no clear pattern in the responses measured apart from the differences observed in genotypic performance. The fast-developing winter cultivar Longsword sown at lower density was the highest yielding treatments at Loxton in 2017 and 2018 irrespective of N management. Whereas at Yarrawonga, the slower developing cultivar DS Bennett sown at higher densities was the higher yielding treatment.

**Figure 5:**
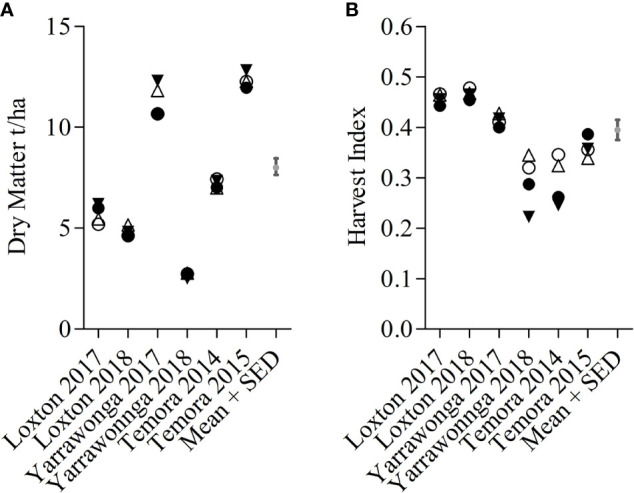
E × CM interactions for DM **(A)** and HI **(B)** at Loxton 2014, Loxton 2015, Yarrawonga 2017, Yarrawonga 2018, Temora 2014 and Temora 2015 for pooled genotypic means. Crop Management factors are ▼ Low Density and Seedbed N, Δ Low Density and Deferred N, ● High Density and Seedbed N, and ○ High Density and Deferred N.

Canopy management × environment interactions were significant for HI and DM across all genotypes however this was largely due to the two stem-frosted sites Temora 2014 and Yarrawonga 2018 (**Figure 5**). Deferred N increased HI at these sites irrespective of planting density. At other sites, there were not any significant differences between canopy management treatments. DM responses varied with environment and the effect sizes were small. DM increased with seedbed N at Loxton in 2017 and lower densities and seedbed N increased DM in favorable conditions at Yarrawonga in 2017 and Temora 2015.

### G × E × Defoliation Responses

In 8 out of 14 G × E combinations, defoliation increased HI, but increased GY in only 2 combinations. GY was similar between treatments at 7 out of 14 combinations and decreased in 5 combinations (**Table 6**). HI was never decreased by defoliation and remained the same as undefoliated controls in the other 6 G × E combinations.

**Table 6:** Mean grain yield (GY), harvest index (HI), above ground dry matter at maturity t/ha (DM), grain number (GN), kernel weight (KW), plant height (Hght) of winter cultivars across environments, and the management effect size when defoliated.

G × E	Control trait means						Management effect (defoliation)					
	GY(t/ha)	HI	DM(t/ha)	GN(no/m^2^)	KW(mg)	Hght(cm)	GY(t/ha)	HI	DM(t/ha)	GN(no/m^2^)	KW(mg)	Hght(cm)
Loxton 2017												
DS Bennett	2.4^b^	0.46^a^	5.8^b^	9922^a^	27.3^c^	65^c^	−0.3	ns	−1.0	−1,064	−1.7	2
Kittyhawk	2.5^b^	0.43^b^	6.9^a^	9050^a^	32.8^b^	73^b^	−0.5	0.04	−2.5	−1,433	−3.9	−13
Longsword	2.5^b^	0.41^c^	7.0^a^	7344^b^	38.4^a^	78^a^	ns	0.09	−1.8	ns	−4.1	−13
Loxton 2018												
DS Bennett	1.6^b^	0.50^a^	4.4^b^	6882^b^	32.2^b^	58^b^	ns	ns	ns	ns	ns	ns
Kittyhawk	1.3^c^	0.42^c^	4.5^b^	4905^b^	38.1^a^	61^a^	ns	ns	ns	ns	1.5	ns
Longsword	2.0^a^	0.47^b^	5.7^a^	7079^a^	37.3^a^	61^a^	ns	0.03	ns	ns	ns	-6
Yarrawonga 2017												
DS Bennett	5.1^a^	0.42^a^	12.1^b^	14795^a^	34.4^b^	98^a^	−0.5	ns	ns	-950	ns	−2
Kittyhawk	4.8^b^	0.41^a^	11.8^b^	12706^b^	39.8^a^	89^b^	−0.6	ns	−2.1	−1728	−1.2	−3
Longsword	4.7^b^	0.36^b^	13.0^a^	11961^b^	39.1^a^	90^b^	−0.3	0.07	−3.3	ns	ns	−10
Yarrawonga 2018												
DS Bennett	1.2^a^	0.38^a^	3.3^a^	3780^a^	34.5^c^	59^a^	ns	0.04	ns	ns	ns	−2
Kittyhawk	0.3^b^	0.11^c^	3.0^a^	912^b^	40.2^a^	58^a^	0.5	0.18	ns	1023	ns	ns
Longsword	0.5^b^	0.17^b^	2.4^b^	1126^b^	37.4^b^	53^b^	0.4	0.2	ns	1314	ns	ns
Temora 2014												
Wedgetail	2.1	0.26					ns	0.07	−1.3			
Temora 2015												
Wedgetail	3.8	0.35					ns	ns	−1.3			

Different letters in superscript within a site indicate significant differences and ns indicates no significant effect of management at the 95% confidence level for each trait relative the control trait.

Where GY was reduced by defoliation this was associated with a reduction in total crop biomass and grain number. It was possible to increase HI in Kittyhawk at Loxton, and in Longsword at Yarrawonga in 2017 but yield decreased due to a large reduction (>2 t/ha) in DM and a combination of reduced grain number and kernel weight. On these occasions reductions in plant height were also greater than 10 cm. Whereas in the example of DS Bennett at Loxton and Yarrawonga in 2017 HI remained similar to the control when defoliated but GY decreased due to a reduction in grain number and either DM or kernel weight and plant height effects were small (± 2–3 cm).

Where defoliated GY were similar to the undefoliated controls such as Loxton in 2018, this was due to the ability of genotypes to maintain grain number. When HI was increased by defoliation and there were small reductions in biomass and or height, and thus grain number remained similar to undefoliated treatments. There were also three combinations were HI and GY were similar and genotypes recovered all their yield without any negative tradeoffs.

Genotypic differences were evident and despite larger reductions in biomass Longsword generally recovered more yield from defoliation than other cultivars. This is reflected in HI as Longsword was the most responsive cultivar to defoliation (increased HI at all sites), whereas DS Bennett was the least responsive but increased HI at the stem frosted site (Yarrawonga 2018), defoliation increased HI in Wedgetail when stem frosted at Temora 2014. At sites where DS Bennett had a greater HI, it always had reduced biomass compared to Longsword meaning yields between cultivars were often similar at high and low HI. Despite the inconsistent effects of management and the strong influence of the environment, genotypic differences in HI were stable and consistent across sites and management. DS Bennett tended to have higher HI than both Kittyhawk and Longsword.

### G × M Interactions Under Frost and Heat Shock

**Figure 6:**
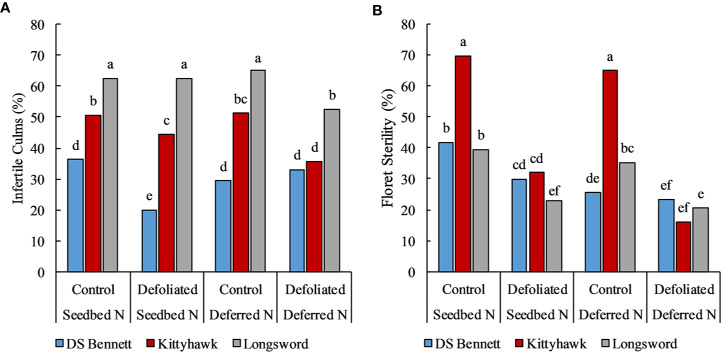
The % of infertile culms **(A)** and % of sterile florets **(B)** in response to management factors deferring Nitrogen (N) and defoliation for winter genotypes DS Bennett, Kittyhawk, and Longsword at Yarrawonga 2018. Different letters indicate significant differences at the 95% confidence level.

**Figure 7:**
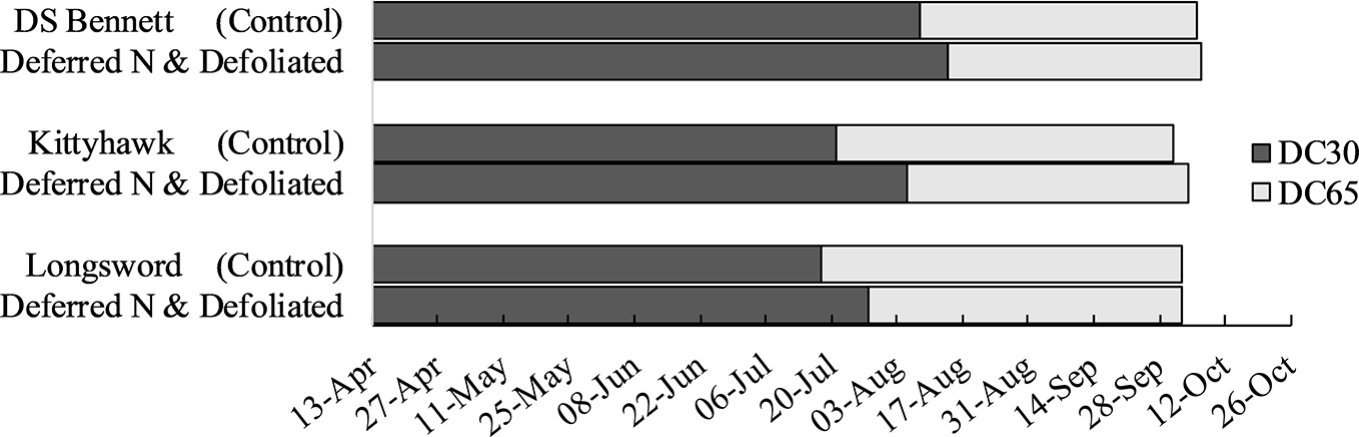
Timing of the onset of stem elongation (DC30), and flowering (DC65) in seedbed applied N control treatments for genotypes DS Bennett, Kittyhawk, and Longsword relative to the management intervention deferring N and defoliation at Yarrawonga 2018.

Stem frost damage was only evident at Temora 2014 and Yarrawonga 2018 when temperatures were below –4°C in August (**Table 3**). At Temora, seeding density and defoliation had no significant effect on the number of infertile stems (data not presented), however deferring N reduced damage by 7% relative to the seedbed N (31% infertile stems). Yield was rarely increased by defoliation but on the occasions it was associated with increased HI and GNO at similar DM to undefoliated controls. This only ever occurred at Yarrawonga in 2018 in the early mid-winter cultivars which were Longsword (+0.5t/ha) and Kittyhawk (+0.4t/ha) which were more by affected by severe stem frost, and defoliation reduced frost damage. At Yarrawonga seeding rate had little impact on stem frost damage, genotypic differences had the largest effect which could be potentially attributed to genetic differences in tolerance, developmental differences and crop architecture. Longsword had the greatest amount of stem damage under all management combinations, Longsword was also the first cultivar to reach DC30 and DC65 (**Figure 7**), followed by Kittyhawk, and DS Bennett. Management effects were significant, compared to seedbed applied N the management intervention of defoliation and deferred N reduced the damage from 63% to 53% in Longsword, from 51% to 36% in Kittyhawk, and from 36% in DS Bennett to 33% which wasn’t significant (**Figure 6**). The same management intervention of deferred N and defoliation applied at Yarrawonga 2018 delayed the timing of stem elongation by 10 days in Longsword, 15 days for Kittyhawk, and 6 days for DS Bennett (**Figure 7**).

Management and genotype had a significant effect on the amount of floret sterility depending on the environment and severity of the damage. The effect of management was not significant at Loxton in 2017 and 2018, and Yarrawonga in 2017. However genotypic differences were significant, Longsword had on average 19% (Lo17), 2% (Lo18), and 7% (Ya17) sterility. Kittyhawk was 12%, 5%, and 5%, whereas DS Bennett always trended lower 11%, 2%, and 1%, respectively. The effect of genotype × management was significant at Ya18, seeding rate was insignificant but defoliation and deferred N reduced the amount of sterility relative to the untreated controls. This management combination reduced floret sterility in DS Bennett from 42% to 23%, from 70% to 16% in Kittyhawk, and 40% to 21% in Longsword (**Figure 6**).

**Figure 8:**
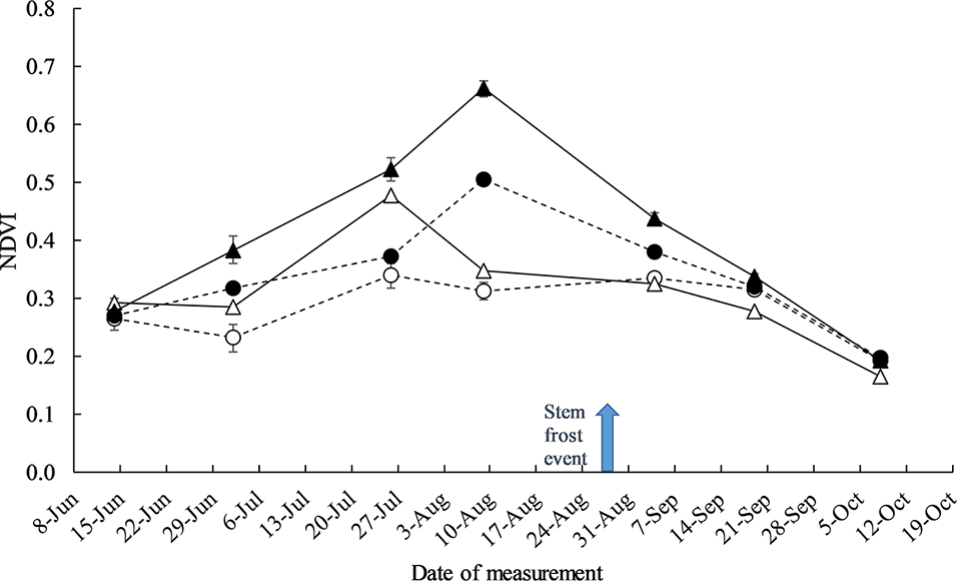
The effect of crop management practices defoliation and deferring N on recorded canopy NDVI measurements at Yarrawonga 2018. Crop management factors are ▲ Control and Seedbed N, ● Control and Deferred N, Δ Defoliated and Seedbed N, and ○ Defoliated and Deferred N. The arrow indicates the timing of a severe stem frost event (−6°C). Data is averaged across all plant density and genotypes, the error bars represent the SED.

Management practices N, defoliation, plant density, and genotype had a significant effect on NDVI and canopy cover at Yarrawonga in 2018 (data not presented). To explain the observations in **Figure 6** of frost damage there was significant differences in canopy structure as measured by NDVI during the timing of severe stem frost. There wasn’t any significant G × defoliation × N timing three way interaction at any time of the year, however defoliation × N timing significantly changed the canopy structure to the largest degree in all three genotypes after defoliation and in the month of August (**Figure 8**).

## Discussion

All crop management strategies evaluated gave variable HI and GY responses with small effect sizes which interacted unpredictably with environment. We conclude that none of the management strategies evaluated here can be used to reliably improve yield in early sown winter cultivars, whereas improved understanding of G × E interactions could reliably increase yield and HI in early sown wheat.

### HI and Yield Responses to G × M in Water Limited Environments

The HI results have shown examples of early sown slow developing cultivars approaching 0.5 (**Figure 1**) which is nearing the values consistently reported in well managed fast developing spring cultivars sown later in autumn such as 0.45 by Flohr et al. (2018a) and the maximum of 0.56 reported by Unkovich et al. (2010). While it was possible in our experiments, HI’s close to 0.5 were not routinely achieved, and when they were it was often negated by a reduction in DM. The data presented here is an improvement compared to HI previously reported at Condobolin and Cowra in NSW from 0.28 to 0.31 for April-sown plots to 0.38–0.42 for June-sown plots (Batten et al., 1999), but there is still significant room for improvement in early sown wheat.

The highest yielding treatments at each environment occurred in cultivars that could maintain high DM and high HI. Defoliation had the most reliable positive effect on HI but also tended to reduce DM accumulation which negated increases in HI resulting in neutral yield responses. These results are consistent with previously published studies which have shown that that defoliation often increases HI of winter crops, but mainly though reduced total shoot biomass rather than increased GY because at maturity, grazed crops typically have lower stem and leaf DM, but spike DM remains similar to controls (Harrison et al., 2011b). Therefore, yield responses to defoliation are variable, but tend to decrease yield compared with un-grazed controls (Harrison et al., 2011a).

Responses to plant density and nitrogen timing were variable and had small effect sizes. Deferring N had very little impact on yield, even comparing the two extremes of canopy management (high plant density and early N compared to low plant density and delayed N). There appeared to be no responses resembling “haying off’ that have been reported in fast developing wheat (Van Herwaarden et al., 1998) to suggest high plant density and early N are leading to excessive vegetative growth and water use. Reducing plant density has also been proposed as a way of reducing early DM accumulation and improving HI in early established slow developing cultivars (Kirkegaard et al., 2014). We found very limited evidence to suggest low plant densities increased HI or yield of early sown wheat suggesting lower plant densities are not saving anymore water for post-anthesis use. This may be due to the capacity of winter wheats to tiller and negate any significant changes in plant density compared to the shorter vegetative period of fast developing wheats. Spike densities were slightly lower at lower plant densities and deferred N applications; however, grain number was maintained by increases in grains per spike in lower density and deferred N treatments. This partially implies that winter cultivars maybe source limited and relatively inefficient at partitioning to spike growth during the critical period. This could suggest a breeding target for improved yield in winter cultivars. Our results add to the body of field experiments outlined in the review of Hunt (2017) that have found no positive effect on yield of reducing plant density in early established winter cultivars across either high, medium, and low water limited yield environments.

The lack of significant large effects of N timing and plant density highlights breeders can select from a broad range of early sown environment × management combinations, and growers have great flexibility in how they manage early sown crops. There is some evidence to suggest deferring N could increase yield in Longsword at Loxton 2018, and in Wedgetail at Temora 2014. There were two instances where defoliation increased HI and yield at Yarrawonga in 2018, due to increased grain number from reduced damage from stem frost in faster developing winter cultivars. These findings nonetheless highlight across a large range of yield environments and stresses that responses to plant density, deferring N, and defoliation are likely to be yield neutral or small and may offer a benefit in frost prone landscapes.

### Improving HI and Yield in Frost Prone Landscapes

The only sites were HI was positively correlated to yield were severely frosted. The G × E × M responses to frost damage observed at Yarrawonga and Temora suggests the severity of stem frost damage can be partially managed with agronomy and genetics to increase HI and yield under these circumstances. Cold temperatures can cause pre-heading stem damage if temperatures approach < −6°C similar to Ya18 and Te14, and if the head emerges after such a frost event, this damage often presents as a bleached section with incomplete ear structure and aborted florets as explained in Frederiks et al. (2015). The improvement in yield, HI, and reduced stem damage from deferring N and grazing in fast mid-developing cultivars Longsword, and Kittyhawk at these sites relative to DS Bennett is likely due to a combination of phenology, and canopy structure. Grazing and deferred N delayed the onset of stem elongation and led to differences in crop canopy structure (**Figure 8**). Differences in crop canopy structure can affect the flow of air movement leading to different heat fluxes in the canopy around the stems and exposed spikes (Gusta and Wisniewski, 2013; Frederiks et al., 2015). Practices that are aimed at reducing the density of the canopy and increasing heat storage bank and thus radiance of the soil have been proposed before (Rebbeck et al., 2007). However, quite often management strategies that reduce or change canopy structure such as defoliation may also lead to decreased yield potential by reducing grain number per unit area as evidenced in DS Bennett at Kittyhawk in sites that were not severely frosted. The faster developing cultivar Longsword managed to maintain grain number at non frosted sites when N was deferred and plants defoliated and increased grain number at frosted sites. This strategy for fast and mid-developing winter cultivars needs further mechanistic investigation.

It is not entirely clear whether the improved stem frost tolerance from defoliation is due to phenological avoidance or a greater thermal insulation from changes in canopy structure. Genetic differences may also exist for frost tolerance but these have not been evaluated in depth here and are limited in the literature (Frederiks et al., 2015). Phenological avoidance or frost escape is a likely explanation for the reduced damage and lack of management responses observed in DS Bennett as it flowers later than other cultivars but still managed to maintain high yields. However, phenological avoidance alone for reproductive frost damage for the fast mid-developing cultivars seems an unlikely explanation as none of the management factors significantly delayed flowering date by more than four days at any site in Longsword and Kittyhawk but there were marked reductions in sterility in response to management.

### Future Improvement in HI and GY Within a G × E × M Framework

With the exceptions of environments that were characterized by severe stem frost, the management factors presented here have shown limited scope to improve HI and yield in early sown crops. Nonetheless, the responses of the cultivars in these series of experiments do suggest future yield gain may be able to be achieved through further increases in partitioning of assimilates to the growing spike that lead to increased grain number (Slafer et al., 2015). In the past, breeders have indirectly selected for lines with reduced pre-flowering senescence that invest an increasing amount of resources toward reserve and reproductive organs, which ultimately translates in greater yield (Sadras and Lawson, 2013). Since yield has largely been improved by indirect selection for higher HI, direct selection for partitioning traits may accelerate HI and GY increases in early sown slow developing wheat (**Box 1**).

Box 1 **Breeding and allometric relationships of cultivars**

Within the context of early sowing our results have demonstrated that crop management practices of nitrogen timing, plant density, and defoliation are unlikely to provide the necessary increases in HI required to further increase yield in early sown crops. The lack of significant relationship observed between HI and GY means a higher HI did not always result in higher grain yield. Qin et al. (2013) concluded that breeders should focus on reproductive allometry of individuals when interpreting HI and select for allometric patterns that are most advantageous in a given agronomic context, especially when there is large variation in productivity among individuals, locations, or years.

Given responses to management were small and inconsistent, it may be more useful to study allometric relationships between DM and GY within genotypes across a broad range of management practices and environment. Using an allometric approach it may be possible to increase HI by selection for high DM and grain yield. In this study, the slope of the relationship between DM and GY was similar for genotypes across all pooled means combining environment and management, meaning the genotypic differences in the ability to convert DM to yields is relatively consistent across a broad range of environments that differ in potential yield and biomass production (**Box Figure 1**). However, intercepts differed significantly between cultivars. Longsword had a significantly (P < 0.01) higher intercept than DS Bennett and Kittyhawk, this means Longsword is partitioning biomass more efficiently than other genotypes in low yielding environments and the slower developing cultivar DS Bennett had a higher HI and yield at Yarrawonga as it partitioned biomass more efficiently at higher yielding environments.

**Box Figure 1:**
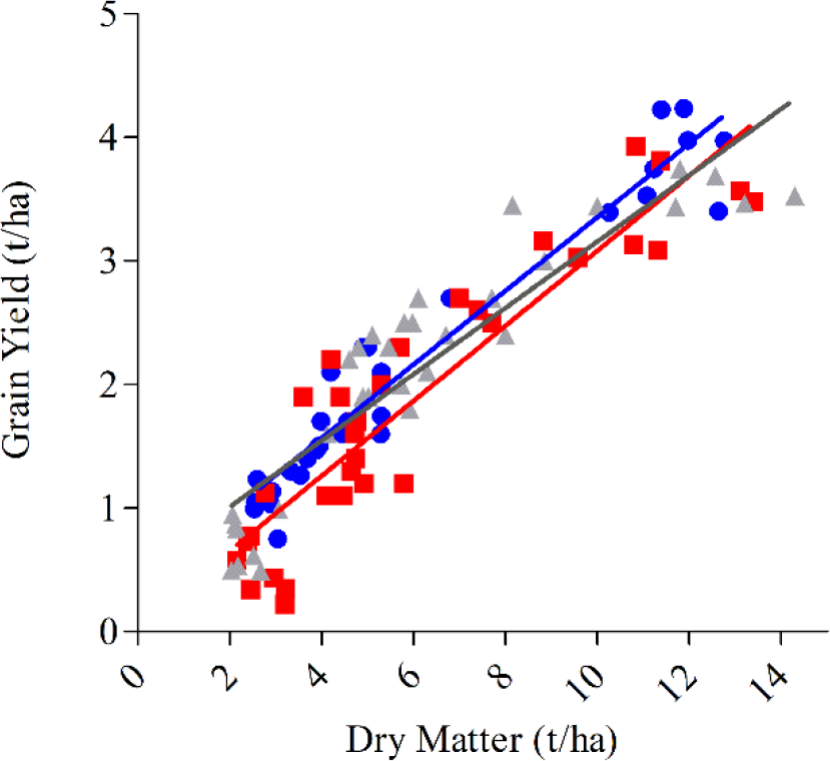
The aboveground biomass at maturity (t/ha) plotted against grain yield (t/ha) for genotypes DS Bennett (● y = 0.30x + 0.37, <0.001, R^2^ = 0.93), Kittyhawk (■, y = 0.30x + 0.043, and <0.001, R^2^ = 0.84) Longsword (▲, y = 0.26x + 0.47, <0.001, R^2^ = 0.84). Data points are contributed by management × environment combinations applied to increase HI within each genotype (n = 32 per genotype).

Monaghan et al. (2001) proposed that the deviation from the regression line between grain yield and grain protein could be used to identify genotypes having a lower or higher protein than expected from their GY. A similar approach could be used for grain yield and dry matter. Using mean deviation values obtained from across a wide range of environments it was possible to identify cultivars that deviated positively or negatively from the regression line regardless of the management factor, showing DM/GY deviation has a genetic basis. Genotypes with a positive deviation consistently yielded higher than those with a negative deviation (**Box Figure 2**). Longsword had a higher mean deviation at Loxton in both seasons and DS Bennett was higher at Yarrawonga meaning they partitioned biomass more efficiently at those respective sites. The negative deviation in the genotype Kittyhawk describes its poor partitioning and it achieves lower grain yield for a given dry matter compared to other varieties.

The G × E interaction observed in these data is likely a function of flowering time and highlights why adapting phasic development to the environment to ensure an optimal flowering time remains one of the critical factors in achieving a high HI in water-limited environments and should not be overlooked in the quest for higher HI through partitioning traits. This enables the crop to produce sufficient biomass by anthesis and minimizes losses in floral fertility from heat, frost and water stress, while leaving sufficient water for grain filling (Passioura and Angus, 2010; Flohr et al., 2018b). The flowering stability and improved biomass potential of cultivars like Longsword and improved grain number potential of DS Bennett pave the way for more genetic progress on other traits strongly correlated to improved HI

**Box Figure 2:**
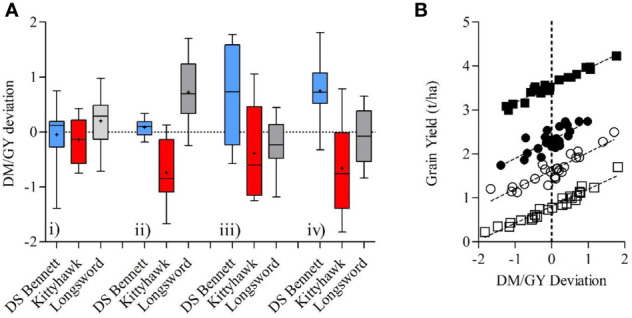
**(A)** Boxplots comparing the variation in the DM/GY residual deviation of three cultivars DS Bennett, Kittyhawk, Longsword over 4 environments Loxton 2017 (i), Loxton 2018 (ii), Yarrawonga 2017 (iii), and Yarrawonga 2018 (iv) contributed by all management practices at each site to increase HI. **(B)** The relationship between the DM/GY deviation and grain yield across all G × M combinations at 4 sites, Loxton 2017 (●, y = 0.42x + 2.32, P < 0.001, R^2^ = 0.64), Loxton 2018 (○, y = 0.41x + 1.62, P < 0.001, R^2^ = 0.83), Yarrawonga 2017 (■, y = 0.39x + 3.56, P < 0.001, R^2^ = 0.96), and Yarrawonga 2018 (□, y = 0.38x + 0.81, P < 0.001 R^2^ = 0.94). (n = 24 per site).

The fast-developing cultivar Longsword’s response to defoliation provided some insight into other strategies that can increase HI and GY. Relative to other cultivars Longsword increased HI and recovered more yield from defoliation (compared to the nil control) in 2017. This was achieved by reducing both DM and height but maintaining grain number. The increased tillering capacity of slow developing wheat could have implications for source sink relationships and increased plant height may not be required. While our experiments fall within the optimal height of 0.7–1 m for modern wheat cultivars proposed by Richards (1992), the same analysis has not been undertaken for early sown winter wheat despite the yield increase observed from shorter wheats (Sayre et al., 1997). Increased plant height and more leaves lead to competition for carbohydrates between the developing spike and elongating stem of early sown crops (Gomez-Macpherson and Richards, 1995). Increasing partitioning to spike growth at the expense of stem and other structural organs (rachis, glumes and palea) within the spike may provide an avenue for the improvement of the HI of wheat (Foulkes et al., 2010). Decreased competition for resources between stem growth and the developing spike may be possible with further reductions in plant height through genetic manipulation or management practices that have less effect on DM accumulation such as plant growth regulators. Further studies should investigate the modulation of WSCs particularly as higher apparent translocation ratio of stem WSC might mitigate yield penalties caused by defoliation (Hu et al., 2019). Remobilization of WSC stored in the stems and leaf structures at the timing of anthesis to the grains contributes to a high HI, especially when carbon assimilates for grain filling are limited by water stress during grain fill (Foulkes et al., 2007) and can be an important source of carbohydrates for grain filling in the absence of post-anthesis stress, indicating the importance of remobilization of WSC to high HI even under favorable conditions such as the UK and higher rainfall zones (Foulkes et al., 2002; Shearman et al., 2005; Zhang et al., 2012).

While flowering time should not be ignored, fine tuning of pre anthesis phases is unlikely to further improve HI, as Flohr et al. (2018a) demonstrated HI was decoupled from phase duration. The genotypes used in this study all flowered within the optimal period for each environment. The faster developing Longsword at Loxton, Mid-developing Wedgetail at Temora, and the slower developing DS Bennett at Yarrawonga as outlined in other experiments at these sites. The breeding interest in HI is important as fast developing cultivars are approaching the upper limit of HI, and future yield gains will have to be sought through increased DM production in spring wheat. Flohr et al. (2018a) proposed future yield gains may be achieved by combining the superior partitioning of modern fast developing cultivars with the longer duration of growth and thus greater DM production of early sown winter cultivars. Further enhancement in HI without compromising DM would increase GY in early sown winter wheat, in the same way it has been achieved in the past with spring wheats, South Australian cultivars released after the early 1980s accumulated more DM (Sadras and Lawson, 2011) and other key studies show that breeding has increased HI with little effect on total DM production (Perry and D’antuono, 1989; Siddique et al., 1989; Calderini and Slafer, 1999; Flohr et al., 2018a). It is acknowledged in the UK that future improvements in yield will require more crop biomass (Mitchell and Sheehy, 2018), there is evidence in the literature of HI in the UK of as high as 0.6 (Shearman et al., 2005) and while we recognize Australian conditions are more water limited than the UK it is therefore feasible to assume that 0.6 could be achieved and falls within theoretical maximums of 0.62 proposed by Austin et al. (1980), 0.64 by Foulkes et al. (2010), and 0.66 by Shearman et al. (2005).

The longer growing period of early sown slow developing wheat would imply that there is the potential to lift wheat yields through increasing DM production and focusing on partitioning traits (**Box 1**) such as HI and fruiting efficiency (Slafer et al., 2015). Early generation direct selection for HI in single spaced plants was proposed by Fischer and Rebetzke (2018) as a means of accelerating increases in potential yield. The breeding effort for winter wheat is still relatively immature in Australia and breeders are still considering the most cost effective phenotyping strategies to increase genetic gain in yield of winter wheat. It is for this reason we believe the method we propose of selecting for a high HI in single plants will accelerate yield gain in Australian winter wheats and should be considered by Australian breeders. Breeders would however need to be conducting screening within the context of the right management as discussed in this study, that is in early sown crops and selecting for appropriate flowering time in addition to selecting for a high HI and partitioning traits.

## Conclusion

Given the limited effect of management strategies found here, we propose that genetic improvement is the most promising avenue for increasing HI and yield in early sown wheat, and postulate that this could be achieved more rapidly through continued selection for phenology adapted to target environments and possibly early generation selection of partitioning traits such as HI and fruiting efficiency.

## Data Availability Statement

The datasets generated for this study are available on request to the corresponding author.

## Author Contributions

KP and JH conceived and designed the experiments. KP, MS, and JH conducted the experiments and performed the data collection. KP performed data analysis and drafted the manuscript. JH made substantial contributions to data analysis and interpretation, as well as manuscript writing. All authors contributed to the article and approved the submitted version.

## Funding

The research undertaken as part of this project is made possible by the significant contributions of growers through both trial cooperation and the support of the GRDC through research project numbers ULA 9175069 and CSP00178. The authors would like to thank them for their continued support.

## Conflict of Interest

The authors declare that the research was conducted in the absence of any commercial or financial relationships that could be construed as a potential conflict of interest.

The handling editor is currently organizing a Research Topic with one of the authors JH.
