# Supplementary material for: Evaluation of G × E × M Interactions to Increase Harvest Index and Yield of Early Sown Wheat
Source: Front Plant Sci. 2020 Jul 10;11:994. doi: 10.3389/fpls.2020.00994 (PMC7366857; doi:10.3389/fpls.2020.00994)
Supplement: Supplementary file 1 [file DataSheet_1.docx]

Supplementary Material

Supplementary Table 1. Percentage of variance accounted for by environment (E) and management factors defoliation (D), nitrogen timing (NT), and plant density (PD) on grain yield, dry matter, and HI in 6 experiments in SE Australia in years 2014 – 2018. Shaded cells are significant at 95% level of confidence

|  | Grain Yield | Dry Matter | Harvest Index |
| --- | --- | --- | --- |
| Environment (E) | 82.0 | 83.9 | 44.3 |
| Defoliation (D) | 0.3 | 2.2 | 6.5 |
| Nitrogen timing (NT) | 0.1 | 0.0 | 1.9 |
| Plant density (PD) | 0.5 | 0.3 | 0.0 |
| E x D | 1.1 | 1.1 | 4.9 |
| E x NT | 1 | 0.2 | 2.4 |
| D x NT | 0.0 | 0.2 | 0.2 |
| E x PD | 1.2 | 0.5 | 0.5 |
| D x PD | 0.0 | 0.1 | 0.1 |
| NT x PD | 0.0 | 0.0 | 0.1 |
| E x D x NT | 0.3 | 0.1 | 0.2 |
| E x D x PD | 0.1 | 0.0 | 0.2 |
| E x NT x PD | 0.1 | 0.1 | 0.9 |
| D x N x PD | 0.1 | 0.1 | 0.0 |
| Residual error | 13.3 | 10.8 | 37.1 |

Supplementary Table 2 Analysis of variance output on the Genotype x Environment x Management interactions for grain yield (GY), dry matter at maturity (DM), harvest index (HI), kernel weight (KW), grain number (GN), spike density (SD), and plant height (PH)

|  | GY | DM | HI | KW | GN | SD | PH |
| --- | --- | --- | --- | --- | --- | --- | --- |
| Environment (E) | *** | *** | *** | *** | *** | *** | *** |
| Genotype (G) | *** | ns | *** | *** | *** | *** | ** |
| Defoliation (D) | *** | *** | *** | *** | ns | *** | *** |
| Canopy Management (CM) | ** | *** | *** | *** | *** | *** | *** |
| G x E | *** | *** | *** | *** | *** | *** | *** |
| E x D | *** | *** | *** | *** | ** | * | *** |
| G x D | ** | * | *** | ns | ns | ns | *** |
| E x CM | *** | *** | *** | * | ** | ns | ns |
| G x CM | ns | ns | ns | * | ns | ns | ** |
| D x CM | ns | ** | ns | ns | * | * | ns |
| G x E x D | ** | ** | *** | ** | ns | ns | *** |
| G x E x CM | ** | ns | ns | ns | ns | ns | ns |

***, significant at P < 0.001; **, significant at P < 0.01; *, significant at P < 0.05; ns, not significant.


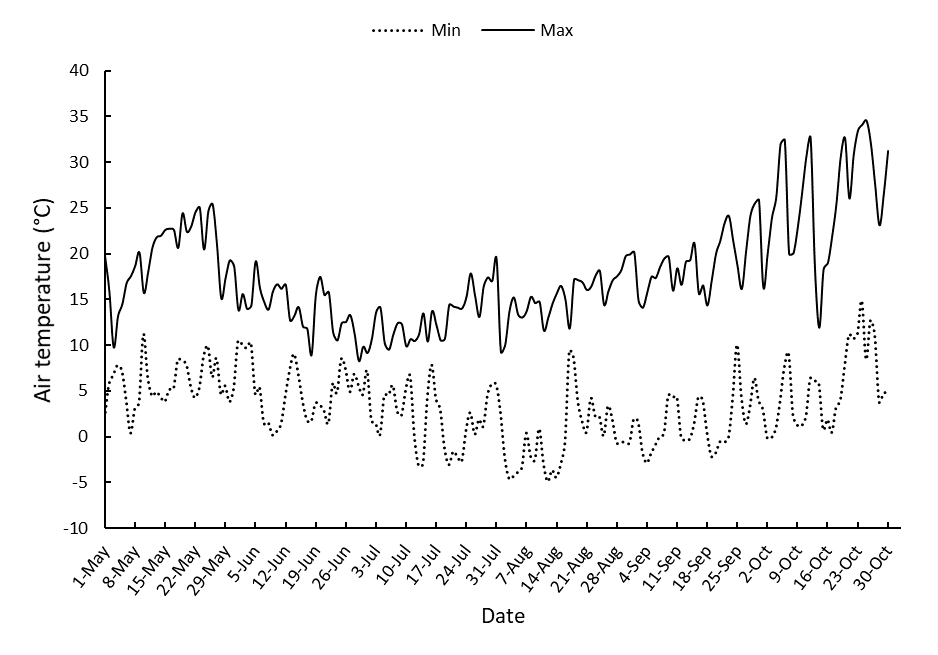


Supplementary Figure 1. Recorded daily air temperature measurements (logged in a screen at 1m) of lowest minimum temperature, and highest maximum temperature during the period from May to November at Temora 2014.


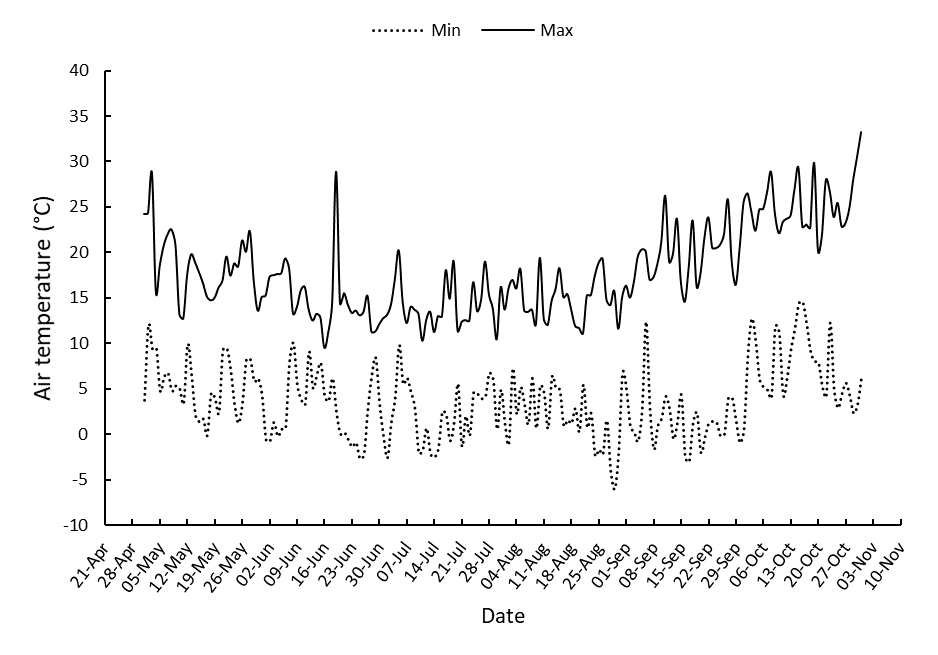


Supplementary Figure 2. Recorded daily air temperature measurements (logged in a screen at 1m) of lowest minimum temperature, and highest maximum temperature during the period from May to November at Yarrawonga 2018 taken in a screen at 1m.
